# Effect of Degradation of Zearalenone-Contaminated Feed by *Bacillus licheniformis* CK1 on Postweaning Female Piglets

**DOI:** 10.3390/toxins8100300

**Published:** 2016-10-17

**Authors:** Guanhua Fu, Junfei Ma, Lihong Wang, Xin Yang, Jeruei Liu, Xin Zhao

**Affiliations:** 1 College of Animal Science and Technology, Northwest A & F University, Yangling 712100, Shaanxi, China; guanhua1220@163.com (G.F.); mamafeiabc@163.com (J.M.); 18710354317@163.com (L.W.); yangx0629@163.com (X.Y.); 2 Institute of Biotechnology and Department of Animal Science and Technology, National Taiwan University, Taipei 10617, Taiwan; jrliu@ntu.edu.tw; 3 Department of Animal Science, McGill University, Montreal, QC H9X 3V9, Canada

**Keywords:** *Bacillus licheniformis* CK1, zearalenone (ZEA), serum hormones, estrogen receptor (ER), post-weaning female piglets

## Abstract

Zearalenone (ZEA), an estrogenic mycotoxin, is mainly produced by *Fusarium* fungi. In this study, *Bacillus licheniformis* CK1 isolated from soil with the capability of degrading ZEA was evaluated for its efficacy in reducing the adverse effects of ZEA in piglets. The gilts were fed one of the following three diets for 14 days: a basic diet for the control group; the basic diet supplemented with ZEA-contaminated basic diet for the treatment 1 (T1) group; and the basic diet supplemented with fermented ZEA-contaminated basic diet by CK1 for the treatment 2 (T2) group. The actual ZEA contents (analyzed) were 0, 1.20 ± 0.11, 0.47 ± 0.22 mg/kg for the control, T1, and T2 diets, respectively. The results showed that the T1 group had significantly increased the size of vulva and the relative weight of reproductive organs compared to the control group at the end of the trial. The T1 group significantly decreased the concentration of the luteinizing hormone (LH) compared with the control and T2 groups. Expression of ERβ was significantly up-regulated in the T2 group compared with the control. In addition, expression of ERβ was not different between the control and the T1 group. In summary, our results suggest that *Bacillus licheniformis* CK1 could detoxify ZEA in feed and reduce the adverse effects of ZEA in the gilts.

## 1. Introduction

Mycotoxins are toxic secondary metabolites produced by a range of fungi, especially from *Fusarium*, *Aspergillus*, and *Penicillium* genera [1]. Mycotoxins pose great risks to the health of animals as well as humans. The ingestion of mycotoxin-contaminated feed by animals results in mycotoxin accumulation in different organs or tissues, endangering animal health or entering into the food chain through meat, milk, or eggs [2]. Humans get directly exposed to mycotoxins as a result of eating contaminated crops, or indirectly exposed by consuming contaminated animal products. In addition, the absorption of mycotoxins can be via respiratory or dermal exposure [3,4,5]. Over 400 mycotoxins have been identified, but thousands of mycotoxins may exist [6]. The number of mycotoxins could be changed according to a newly proposed definition of mycotoxins [7]. The new definition states that something is a mycotoxin if and only if it is a secondary metabolite produced by microfungi, posing a health hazard to human and vertebrate animal species by exerting a toxic activity on human or vertebrate animal cells in vitro with 50% effectiveness levels <1000 μM [7]. Of these, zearalenone (ZEA) is one of the most important mycotoxins for its global incidence and toxicity [8].

Zearalenone—a phenolic resorcyclic acid lactone—has good thermal stability and low solubility in water, but is highly soluble in organic solvents. Zearalenone is particularly toxic to the reproductive system, resulting in uterine enlargement, alterations to the reproductive tract, reduced litter size, increased embryo lethal resorption, decreased fertility, and changed progesterone (PRG) and estradiol (E2) plasma levels in laboratory animals [9]. ZEA and 17β-estradiol have similar structures, and both competitively bind to estrogen receptors (ERs). ZEA can activate the transcription of estrogen-responsive genes [10,11]. The estrogenic effects of ZEA are particularly pronounced in the reproductive system of pigs [12].

The Food and Agriculture Organization of the United Nations (FAO) has estimated that approximately 25% of the world’s agricultural products are contaminated with mycotoxins, resulting in significant economic loss due to their impact on human health, trade, and animal productivity [13]. Streit et al. analyzed 13,578 samples of feed and feed raw materials for contamination with ZEA from all over the world over a period of eight years (January 2004–December 2011), and found that 36% of samples were positive for ZEA [14]. Among the positive samples, the average concentration and the maximum concentration were up to 101 μg/kg and 26,728 μg/kg, respectively [14]. Thus, detoxification strategies for contaminated feeds for animals are needed to reduce or eliminate the toxic effects of ZEA in order to improve food safety, prevent economic losses, and reclaim contaminated products. Numerous physical and chemical detoxification methods have been tried, including chemical, physical, and biological approaches. Among them, biological transformations (including the use of microorganisms to breakdown ZEA) are the least studied and may provide an effective means to manage this mycotoxin. Microorganisms in the *Bacillus* genus are considered as probiotics and have been shown to effectively degrade ZEA in vitro. For example, Tinyiro et al. found that *B. subtilis* 168 and *B. natto* were efficient in the removal of more than 75% of ZEA from the liquid medium [15], whereas Cho et al. reported that a *B. subtilis* strain degraded 99% of ZEA in the liquid medium [16]. Moreover, Yi et al. isolated a strain of *Bacillus licheniformis* CK1 from soil samples and found that this strain was capable of degrading ZEA [17]. However, there was limited investigation on feeding animals with microbiologically-detoxified diets [18]. Therefore, the purpose of this study was investigate effects of *Bacillus licheniformis* CK1 on growth performance, vulva size, relative weights of organs, and serum hormone of female piglets fed feed contaminated with ZEA. In addition, we also evaluated the expression of the estrogen receptors in the vagina, uterus, and ovary of the piglets.

## 2. Results

### 2.1. Growth Performance

In the seven-day adaption period, there was no significant difference in the average daily feed intake (ADFI), average daily gain (ADG), or feed efficiency (FE, feed intake/gain) among the three groups. Similarly, during the 14-day feeding period, treatments T1 and T2 exhibited no negative effect on the ADFI, ADG, or FE in comparison with the control (Table 1).

### 2.2. Vulva Size

**Figure 1:**
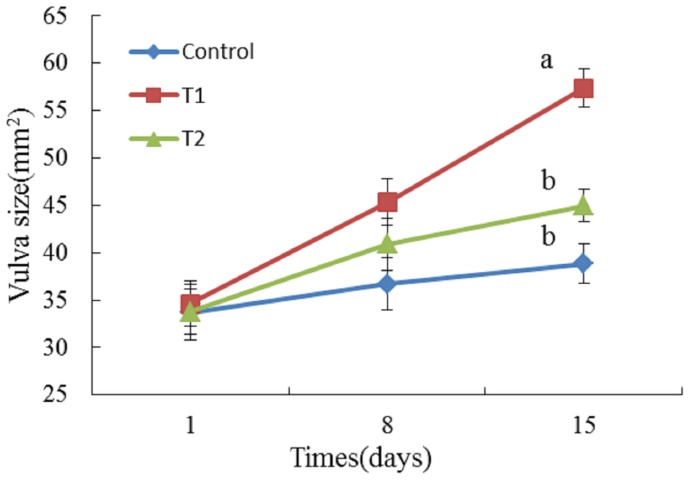
Effects of different diets on the vulva size of female piglets. a, b Values followed by different superscript letters differ significantly (*p* < 0.05, *n* = 6). Control group: the basal diet; T1 group: zearalenone (ZEA)-contaminated diet; T2 group: fermented ZEA-contaminated basic diet by *Bacillus licheniformis* CK1.

**Figure 2:**
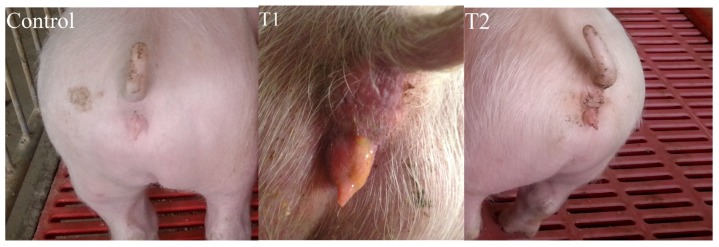
The representative vulva of piglets at the end of the study. (**Control group**) the basal diet; (**T1 group**) zearalenone-contaminated diet; (**T2 group**) fermented ZEA-contaminated basic diet by *Bacillus licheniformis* CK1.

Figure 1 shows changes in vulva size in the piglets in the three groups of piglets. There was no significant difference in the vulva size among the three groups at the beginning of the trial (d1). At the end of the trial (d15), the size of the vulva was significantly increased in the T1 group, but not in the T2 group, in comparison with the control (*p* < 0.05). The vulvae of the piglets in the T1 group were slightly red and swollen. On the other hand, no obvious change was observed in the control and T2 groups (Figure 2).

### 2.3. Organ Weight

The relative organ weight was calculated as the weight of the organs divided by the body weight (g/kg). As shown in Table 2, the relative weight of the liver was significantly lower in piglets in the T1 group compared with the control (*p* < 0.05), while there was no significant difference between the T2 group and the control (*p* > 0.05). Piglets in the T2 group had significantly increased kidney weight, in contrast with the control and the T1 groups (*p* < 0.05). The T1 group and the T2 group had significantly heavier reproductive organs than the control (*p* < 0.05), but the relative weights of reproductive organs in T1 and T2 groups were not different (*p* > 0.05). For the other organs (heart, spleen, and lung), there were no differences among the three diet groups (*p* > 0.05).

### 2.4. The Level of Serum Hormones

The levels of serum hormones at the end of the test period are presented in Table 3. No significant differences were found in the level of follicle stimulating hormone (FSH), estradiol (E2), prolactin (PRL), progesterone (PRG) and testosterone (T) among the three treatments (*p* > 0.05). On the other hand, the T1 diet significantly decreased the concentration of luteinizing hormone (LH) in comparison with the control (*p* < 0.05). The T2 diet ameliorated the effect of ZEA and the levels of LH in the control and T2 groups were similar.

### 2.5. Estrogen Receptor α (ERα) and Estrogen Receptor β (ERβ) mRNA Expression

**Figure 3:**
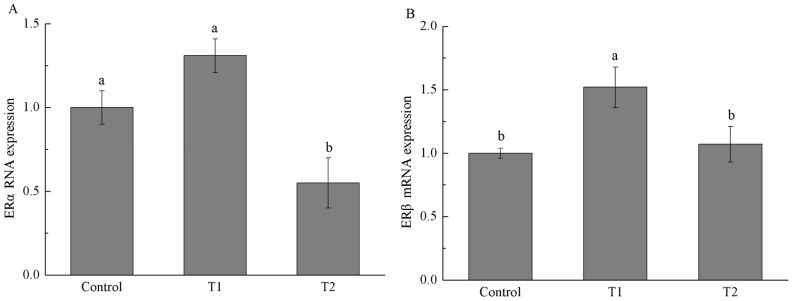

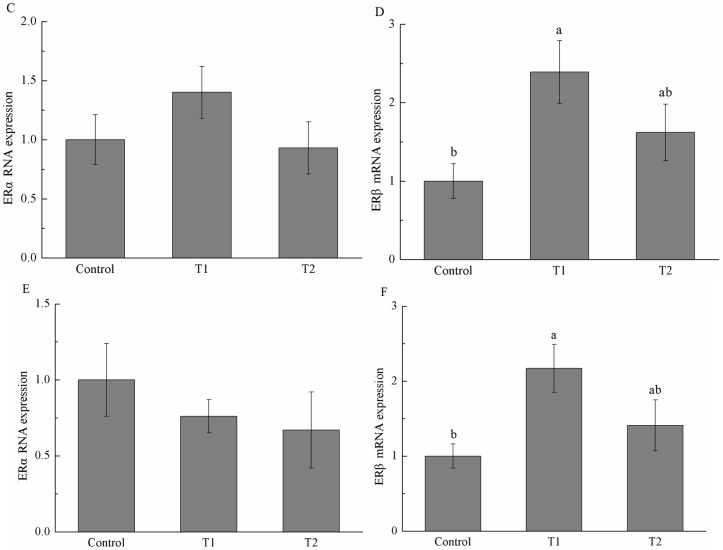
Effects of different diets on the mRNA expression of estrogen receptor α (ERα) ((**A**) vagina, (**C**) uterus, and (**E**) ovary) and estrogen receptor β (ERβ) ((**B**) vagina, (**D**) uterus, and (**F**) ovary) in the reproductive organs of female weaned piglets. Control group: the basal diet; T1 group: zearalenone-contaminated diet; T2 group: fermented ZEA-contaminated basic diet by *Bacillus licheniformis* CK1. ^a, b^ Different letters in each panel indicate significant differences (*p* < 0.05, *n* = 6).

As shown in Figure 3, mRNA expression of ERα and ERβ (two subtypes of estrogen receptor) was quantified in the reproductive organs by real-time quantitative polymerase chain reaction (RT-qPCR). No significant difference in ERα mRNA expression was found in the uterus and ovary among the three treatments, while the T2 group significantly decreased the mRNA expression of ERα in vagina (*p* < 0.05) in comparison with the control and the T1 group. The mRNA abundance of ERβ in the uterus, vagina, and ovary was significantly higher in gilts on the T1 diet. Meanwhile, ERβ mRNA expression in the uterus, vagina, and ovary in the T2 group was not significantly different from those in the control group.

## 3. Discussion

Zearalenone (ZEA) activates estrogen receptors and induces functional and morphological alteration in reproductive organs. The susceptibility to the adverse effect of ZEA is species–dependent, and pigs—especially prepubertal gilts—are very sensitive to ZEA due to its high alpha-hydroxylation activity and low glucuronidation activity [19]. ZEA is a substrate for α and β hydroxysteroid dehydrogenases, which convert ZEA into two stereoisomeric metabolites, α-zearalenol and β-zearalenol. Alpha-hydroxylation results in an increase in estrogenic potency as compared to the parent compound and beta-hydroxylation product [20]. The glucuronidation conjugates metabolites of ZEA hydroxylation and eliminates them through urine and bile fluid. Pigs have a rather low activity of glucuronidation [21].

*Bacillus licheniformis (B. licheniformis)* CK1 efficiently degraded ZEA in the basal diet. The concentration of ZEA in diets offered to the T2 group was reduced to 0.47 mg/kg from 1.20 mg/kg in the T1 group due to degradation by *B. licheniformis* CK1. The reduction of ZEA from 1.20 to 0.47 mg/kg in feed relieved red and swollen symptoms in the vulva of the piglets, and significantly decreased the vulva size of the piglets. Jiang et al. [22] reported the vulva size and relative weight of genital organs, liver, and kidney increased linearly in a ZEA-dose-dependent manner, indicating that the estrogenic effects are stronger with increasing concentration of ZEA. Thus, decreasing the concentration of ZEA in feeds could reduce the estrogenic effects of ZEA in the gilts. In addition, it has been previously shown that *B. licheniformis* CK1 decreased more than 98% of ZEA in ZEA-contaminated corn meal medium and was non-hemolytic, non-enterotoxin producing, and displayed high levels of extracellular xylanase, cellulase, and protease activities [17]. The presence of interfering substance in the basal diet might explain the lower efficiency of *B. licheniformis* CK1 to degrade ZEA in the current study, in comparison with the study of Yi et al. [17].

Whether *B. licheniformis* CK1 could reduce the adverse effects of ZEA for piglets was investigated in the feeding trial. Our results showed that feeding the ZEA-contaminated diet (T1 group) significantly increased the vulva size of gilts. In addition, organ weights were used as an index of estrogenic response to ZEA, especially for reproductive organs. In our study, we observed that the relative weight of reproductive organs in the T1 group was significantly increased compared to the control group. Our results are in agreement with previous reports. Oliver et al. reported that gilts fed ZEA-contaminated diets significantly increased vulva width and length compared with control [23]. Similarly, the vulva width, length, and area of piglets linearly increased as ZEA levels increased [24]. In addition, the relative weight of genital organs was also increased in female piglets supplemented with 1.05 mg/kg ZEA [24]. While there was no significant difference between T1 and T2 groups for the relative weight of genital organs, the T2 group significantly reduced the vulvar swelling of piglets in this study, implying that *B. licheniformis* CK1 can effectively alleviate the estrogen acting on the vulva of postweaning piglets caused by ZEA. Others have reported similar effects by using adsorbent materials or chemicals to deal with ZEA contaminations. Jiang et al. reported that clay enterosorbent at the levels of 5 or 10 g/kg was able to reduce the estrogenic effect of ZEA on vulvar swelling in postweaning female pigs [25]. Moreover, Denli et al. demonstrated that activated diatomaceous clay could effectively spare the estrogenic effect of ZEA on uterus and ovaries in rats and pigs [26]. The addition of a modified calcium montmorillonite alleviated some of the reproductive effects of ZEA on the relative weight of genital organs in postweaning piglets [24,27].

ZEA and its metabolites can be regarded as endocrine disruptors that change hormonal activity at the pre-receptor level. In the current study, ZEA decreased the level of luteinizing hormone (LH) in post-weaning gilts, but had no influence on the level of follicle stimulating hormone (FSH), estradiol (E2), prolactin (PRL), progesterone (PRG), or testosterone (T). Wang et al. observed that ZEA decreased the levels of E2 and LH in pre-pubertal gilts, but had no effect on the level of FSH [28]. Other researchers also reported that serum LH in gilts was significantly decreased by adding ZEA in feeds [29,30]. The gilts in the T2 group had similar LH concentrations to those in the control group, indicating that *B. licheniformis* CK1 has a protective effect on ZEA toxicosis symptoms in piglets.

ZEA and some of its metabolites have been shown to competitively bind to estrogen receptors (ERα and ERβ) in a number of in vitro and in vivo systems [31]. Therefore, we also investigated the mRNA expression level of estrogen receptors in different tissues of piglets among the three groups. In the present study, ERβ expression was significantly increased in the uterus, vagina, and ovary of gilts in the T1 group compared with the control group, whereas ERα was not significantly different. Our results are in agreement with a previous study by Oliver et al. [23]. ERβ could directly bind and accelerate the expression of adipogenic genes, enhancing triglyceride concentrations in ERβ-positive cells [32]. Thus, increasing the size and weight of the reproductive organs in our study—at least in part—could result from altering the expression of ERβ and the subsequent expression of other genes. Contrary to our results, Dong et al. found that the expression of ERα was significantly increased in the uterus of goats by ZEA, while the expression of ERβ was not changed [33]. The different results could be due to the species difference. Expression of ERβ in vagina, uterus, and ovary in the T2 group was similar to those in the control group, but was significantly lower than that in vagina of the T1 group.

In our study, the average daily feed intake (ADFI), average daily gain (ADG), and feed efficiency (FE) of the piglets were not different among the three groups. Likewise, Jiang et al. reported that there was no obvious difference in the growth performance of gilts between the control diet and the diet with ZEA concentration in the range of 1.1 to 3.2 mg/kg. Another study also showed that average daily feed intake did not differ between gilts consuming the control and zearalenone diets, which resulted in similar feed efficiency [23].

During the microbial transformation of ZEA, both estrogenic and non-estrogenic intermediates and by-products can be produced—for example, estrogenic α-zearalenol and β-zearalenol [34,35,36]. Certainly, some microbes could degrade ZEA to non-estrogenic products. For example, *Clonostachys rosea* IFO 7063 was effectively capable of converting ZEA to a non-estrogenic compound, 1-(3,5-dihydroxy-phenyl)-10′-hydroxy-1′E-undecene-6′-one, determined by 2D NMR spectroscopy [37]. The yeast strain *Trichosporon mycotoxinivorans* was also able to decarboxylate ZEA [38] and produce a compound identified as (5*S*)-5-({2,4-dihydroxy-6-[(1E)-5-hydroxypent-1-en-1-yl]benzoyl}oxy) hexanoic acid via NMR spectroscopy [39]. In addition, although the degradation product was not clear, Kriszt et al. [40] reported non-pathogenic *Rhodococcus pyridinivorans* K408 degraded 87.21% ZEA and reduced 81.75% of estrogenic effects. Our results supported that *B. licheniformis* CK1 degraded ZEA and reduced its estrogenic effects, possibly because ZEA was converted to non-estrogenic or less estrogenic compounds.

In conclusion, *B. licheniformis* CK1 could degrade the ZEA in feed and alleviated the adverse effect of ZEA for piglets. Our results support the notion that microbiological detoxification is suitable for the decontamination of mycotoxins in feed with high efficiency, strong specificity, and no environmental pollution [41].

## 4. Materials and Methods

### 4.1. Strains and Chemicals

*Bacillus licheniformis* CK1 was isolated from the National Taiwan University [17]. Purified zearalenone (ZEA), acetonitrile, and methanol (HPLC (high-performance liquid chromatography) grade) were purchased from Sigma-Aldrich (St. Louis, MO, USA). All other chemicals used were of analytical grade.

### 4.2. Preparation of the Experimental Diets

ZEA (47 mg) was dissolved in acetic ether and then mixed with talcum powder. A ZEA premix was prepared by blending ZEA-contaminated talcum powder with 3 kg of the basic diet (Table 4). We prepared two batches of ZEA premixes. One was used in mixing with the basic diet as treatment 1 (T1), which was calculated for a ZEA concentration of 1 mg/kg. The other was used for fermentation by *Bacillus licheniformis* CK1 to degrade ZEA before mixing with the basic diet as treatment 2 (T2).

For the fermentation, batches of 300 g of autoclaved ZEA-contaminated feed were mixed with 2700 mL sterilized water in a 5 L fermentor. The mixture was inoculated with 1% of an overnight bacterial culture of *Bacillus licheniformis* CK1 and incubated at 37 °C, 300 rpm for 36 h. The fermented feed was poured into a basin. To absorb water, the basic diet was gradually added. The mixture was dried at room temperature. A total of 3 kg ZEA-contaminated feed were fermented.

All diets were prepared at the same time and stored in covered containers before feeding.

### 4.3. Determination of ZEA in Feed by HPLC

The concentration of ZEA in feed was determined by high-performance liquid chromatography (HPLC) performed on HPLC instrument including a LC-20AT delivery system (Shimadzu, Kyoto, Japan), a CBM-20A system controller (Shimadzu, Kyoto, Japan), a SIL-20A autosampler (Shimadzu, Kyoto, Japan), a RF-10AXL fluorescence detector (Shimadzu, Kyoto, Japan), and an Ascentis C18 HPLC column (Sigma-Aldrich, Bellefonte, PA, USA; 5 μm particle size, L × I.D. 250 mm × 4.6 mm). The injection volume for quantifying ZEA was 20 μL. The mobile phase consisted of methanol:water 80:20 (*v*/*v*) at a flow rate of 0.5 mL·min^−1^. The detector was set at excitation and emission wavelengths of 225 nm and 465 nm, respectively. A standard curve was established by analyzing six ZEA standard solutions (0.125, 0.25, 0.5, 1, 2, 5 μg/mL), and each concentration was determined in triplicate. The linear regression equation of the standard curve showed an *R*^2^ value >0.99. Before the HPLC analysis, ZEN in the feed was extracted and cleaned up using the Romer Mycosep 226 column (Romer Labs Inc., Union, MO, USA) according to the manufacturer′s instructions. The levels of ZEA in feeds were calculated by using the linear regression equation of the standard curve.

### 4.4. Experimental Design and Animals

A total of 18 post-weaning female piglets (Landrace × Yorkshire × Duroc) weaned at d30 with an average body weight (BW) of 8.19 ± 0.32 (mean ± SE) kg were used in this study. The animal protocols used in this work were evaluated and approved by Institutional Animal Care and Use Committee of Northwest A&F University (Identification code: NWAFAC2014, Date of approval: 16 August 2014). Gilts were randomly allocated to three treatments, with six gilts in each group according to BW. All animals were on the basic diet during a 7 day adaptation period after weaning. The nutrient concentrations of the basic diet met or exceeded minimal requirements according to the National Research Council (NRC) [42]. Pigs were fed the basic diet (control), treatment 1 diet (T1), or treatment 2 (T2) diet during a 14 day test period. The actual ZEA contents (analyzed) were 0, 1.20 ± 0.11, 0.47 ± 0.22 mg/kg for the control, T1, and T2 groups, respectively.

During a 14 day test period, animals were housed individually in metal pens on the Northwest A&F University farm. Throughout the study, animals had free access to feed and water, and room temperature was 26–28 °C. Body weights were measured weekly. Feed intake of each treatment was recorded daily. Vulva length and width were measured on d1, d8, and d15 after treatments started to determine the dietary ZEA estrogenic effects, and the vulva area was calculated approximately as a diamond shape ((vulva length × vulva width)/2) according to Jiang et al. [25].

### 4.5. Sample Collection

Pigs were fasted for 12 h at the end of the experimental period. Blood samples of approximately 10 mL were collected from the jugular vein of all animals into non-heparinized tubes, incubated at 37 °C for 2 h, centrifuged at 1500 × *g* for 10 min at room temperature, and the serum was separated and stored in 1.5 mL Eppendorf tubes at −20 °C for hormone analyses (described below). After collection of blood samples, piglets were immediately euthanized and genital organs (ovary + cornu uteri + vagina − vestibule), liver, kidney, heart, lung, and spleen were isolated, weighed, and examined for gross lesions. Samples of uterus, vagina, and ovary tissue were kept at −80 °C until extraction of total RNA for expression of the ERα and ERβ. Organ weights were expressed on a relative body weight basis (g/kg).

### 4.6. Serum Hormone Analysis

Serum samples were analyzed for follicle stimulating hormone (FSH), luteinizing hormone (LH), estradiol (E2), prolactin (PRL), progesterone (PRG), and Testosterone (T) using commercial radioimmunoassay kits obtained from Tianjin Jiuding medical bioengineering CO., Ltd. (Tianjin, China). All the samples were determined by the Yangling Demonstration Zone Hospital (Yangling, Shaanxi, China).

### 4.7. Total RNA Extraction and Real-Time Quantitative RT-PCR (qRT-PCR)

Total RNA was extracted from frozen tissues using a total RNA Kit from Omega (Norcross, GA, USA), according to the manufacturer’s instructions. The purity of total RNA was ascertained by the A260/A280, and the integrity of total RNA was checked by agarose gel electrophoresis. Total RNA for each sample was converted into cDNA using TaKaRa PrimeScriptTM RT Reagent Kit (TaKaRa Biotechnology CO., Ltd., Dalian, China) according to the manufacturer’s instructions and used for real-time quantitative polymerase chain reaction (RT-qPCR).

A SYBR^®^ Premix Ex Taq kit (TaKaRa Biotechnology CO., Ltd., Dalian, China) was used to measure mRNA expression of estrogen receptor genes (ERα and ERβ) with glyceraldehyde-3-phosphate dehydrogenase (GAPDH) as an endogenous control. Pig-specific primers were designed from published GenBank sequences (Table 5). All of the PCR reactions were performed in triplicate. The relative gene expression levels were determined using the 2^−ΔΔ*C*t^ method [43].

### 4.8. Statistical Analysis

Data were analyzed through ANOVA and Duncan’s multiple range tests using SPSS 16.0 statistical software (SPSS 16.0 Inc., Chicago, IL, USA, 2008). The values are expressed as mean ± S.E. Differences were considered significant at *p* < 0.05.

## Figures and Tables

**Table 1:** Growth performance of piglets fed different diets.

Groups	Average Daily Feed Intake (kg)	Average Day Gain (kg)	Feed Conversion Rate
Control	0.71 ± 0.01	0.40 ± 0.08	1.73 ± 0.09
T1	0.68 ± 0.02	0.42 ± 0.05	1.63 ± 0.05
T2	0.63 ± 0.04	0.39 ± 0.06	1.65 ± 0.09
*p* value	0.289	0.463	0.713

Control group: the basal diet; T1 group: Zearalenone-contaminated diet; T2 group: fermented ZEA-contaminated basic diet by *Bacillus licheniformis* CK1. Values are expressed in mean ± S.E. (standard error, *n* = 6).

**Table 2:** Relative weight of organs in weaned piglets fed different diets.

Group	Relative Weight (g/kg)					
	Heart	Liver	Spleen	Lung	Kidney	Reproductive Organs
Control	4.98 ± 0.24	27.51 ± 1.08 ^a^	2.09 ± 0.30	11.68 ± 0.77	5.32 ± 0.28 ^a^	0.47 ± 0.04 ^a^
T1	4.87 ± 0.10	24.33 ± 0.85 ^b^	1.79 ± 0.07	10.38 ± 0.20	5.24 ± 0.26 ^a^	0.66 ± 0.06 ^b^
T2	5.00 ± 0.17	25.53 ± 0.76 ^ab^	2.04 ± 0.19	11.16 ± 0.42	6.80 ± 0.52 ^b^	0.63 ± 0.04 ^b^
*p* value	0.888	0.048	0.559	0.243	0.015	0.006

Control group: the basal diet; T1 group: zearalenone-contaminated diet; T2 group: fermented ZEA-contaminated basic diet by *Bacillus licheniformis* CK1. Values are expressed in mean ± S.E. (*n* = 6). ^a, b^ Means with different superscripts within same column differ significantly (*p* < 0.05).

**Table 3:** The level of serum sex hormones of the female weaned piglets fed different diets on d15.

Group	Follicle Stimulating Hormone (FSH), mIU/mL	Luteinizing Hormone (LH), mIU/mL	Estradiol (E2), pg/mL	Prolactin (PRL), ng/mL	Progesterone (PRG), ng/mL	Testosterone (T), ng/dL
Control	13.33 ± 0.56	8.68 ± 0.11 ^a^	21.27 ± 1.18	12.64 ± 0.48	0.80 ± 0.14	32.07 ± 2.97
T1	12.55 ± 0.56	8.02 ± 0.12 ^b^	24.38 ± 1.01	12.60 ± 0.72	0.91 ± 0.2	31.85 ± 3.59
T2	12.76 ± 0.26	8.70 ± 0.13 ^a^	23.32 ± 1.57	11.09 ± 0.42	1.13 ± 0.32	31.85 ± 0.96
*p* value	0.521	0.002	0.256	0.124	0.635	0.998

Control group: the basal diet; T1 group: zearalenone-contaminated diet; T2 group: fermented ZEA-contaminated basic diet by *Bacillus licheniformis* CK1. Values are expressed in mean ± S.E. (*n* = 6). ^a, b^ Means with different superscripts within same column differ significantly (*p* < 0.05).

**Table 4:** Ingredients and compositions of the basic diet.

Ingredients	Percentage, %	Nutrients	Analyzed Values
Corn	53.00	Gross energy (MJ/kg)	17.12
Wheat middling	5.00	Crude protein (%)	19.40
Whey powder	6.50	Calcium (%)	0.84
Soybean oil	2.50	Total phosphorus (%)	0.73
Soybean meal	24.76	Lysine (%)	1.36
Fish meal	5.50	Methionine (%)	0.46
l-Lysine HCl	0.30	Sulfur amino acid (%)	0.79
dl-Methionine	0.10	Threonine (%)	0.90
l-Threonine	0.04	Tryptophan (%)	0.25
Calcium phosphate	0.80	-	-
Limestone, pulverized	0.30	-	-
Sodium chloride	0.20	-	-
Premix ^1^	1.00	-	-
Total	100	-	-

^1^ Supplied per kg of diet: vitamin A, 3300 IU; vitamin D3, 330 IU; vitamin E, 24 IU; vitamin K3, 0.75 mg; vitamin B1, 1.50 mg; vitamin B2, 5.25 mg; vitamin B6, 2.25 mg; vitamin B12, 0.02625 mg; pantothenic acid, 15.00 mg; niacin, 22.5 mg; biotin, 0.075 mg; folic acid, 0.45 mg; Mn, 6.00 mg; Fe, 150 mg; Zn, 150 mg; Cu, 9.00 mg; I, 0.21 mg; Se, 0.45 mg.

**Table 5:** Nucleotide sequences of primers for quantitative real-time polymerase chain reaction (qRT-PCR).

Gene	Forward Primer and Reverse Primer (from 5′ to 3′)	Size (bp)	Genbank No.
GAPDH	CCTGGCCAAGGTCATCCATG	500	NM_214220.1
	CCACCACCCTGTTGCTGTAG		
ERα	TTGCTTAATTCTGGAGGGTAC	110	EF195769.1
	AGGTGGATCAAGGTGTCTGTG		
ERβ	GCTCAGCCTGTACGACCAAGTGC	138	NM_001001533.1
	CCTTCATCCCTGTCCAGAACGAG		

GADPH: glyceraldehyde-3-phosphate dehydrogenase.
